# A Highly Efficient Electromagnetic Wave Absorption System with Graphene Embedded in Hybrid Perovskite

**DOI:** 10.3390/mi14081611

**Published:** 2023-08-16

**Authors:** Haitao Yu, Hui Liu, Yao Yao, Ziming Xiong, Lei Gao, Zhiqian Yang, Wenke Zhou, Zhi Zhang

**Affiliations:** 1 Field Engineering College, Army Engineering University of PLA, Nanjing 210007, China; 2 Unit of 32399 of PLA, Nanjing 211131, China; 3 State Key Laboratory for Disaster Prevention & Mitigation of Explosion & Impact, Army Engineering University of PLA, Nanjing 210007, China; 4 Position Engineering Research Office, Army Engineering University of PLA, Nanjing 210007, China; 5 Electromagnetic Environmental Effects Laboratory, Army Engineering University of PLA, Nanjing 210007, China

**Keywords:** organic–inorganic hybrid perovskite, graphene, electromagnetic absorption, conductive network

## Abstract

To cope with the explosive increase in electromagnetic radiation intensity caused by the widespread use of electronic information equipment, high-performance electromagnetic wave (EMW)-absorbing materials that can adapt to various frequency bands of EMW are also facing great demand. In this paper, CH_3_NH_3_PbI_3_/graphene (MG) high-performance EMW-absorbing materials were innovatively synthesized by taking organic–inorganic hybrid perovskite (OIHP) with high equilibrium holes, electron mobility, and accessible synthesis as the main body, graphene as the intergranular component, and adjusting the component ratio. When the component ratio was 16:1, the thickness of the absorber was 1.87 mm, and MG’s effective EMW absorption width reached 6.04 GHz (11.96–18.00 GHz), achieving complete coverage of the Ku frequency band. As the main body of the composite, CH_3_NH_3_PbI_3_ played the role of the polarization density center, and the defects and vacancies in the crystal significantly increased the polarization loss intensity; graphene, as a typical two-dimensional material distributed in the crystal gap, built an efficient electron transfer channel, which significantly improved the electrical conductivity loss strength. This work effectively broadened the EMW absorption frequency band of OIHP and promoted the research process of new EMW-absorbing materials based on OIPH.

## 1. Introduction

With the extensive use of electronic equipment in human life, the electronic equipment used in various frequency bands is increasingly abundant [1,2,3]. In order to deal with the electromagnetic interference and radiation caused by electronic equipment with different electromagnetic frequency bands, it is more urgent to study new EMW-absorbing materials with wide influential electromagnetic frequency bands [4,5,6]. In previous work, it has been proved that organic–inorganic hybrid perovskite (OIHP) is a potential new EMW absorbing material [7,8,9,10]. Still, it has some problems, such as a high doping ratio, thick absorbing body thickness, narrow adequate EMW absorption frequency bandwidth, and low EMW absorption intensity [11]. These problems limit the further application of it in the field of EMW-absorbing materials.

As a typical two-dimensional carbon material, graphene has received much attention and applications in the fields of new energy, photoelectric devices, and high-resistance materials due to its extremely low resistivity, high light transmittance, and excellent mechanical properties [12,13,14,15]. Graphene also attracted wide attention in EMW absorption due to its outstanding dielectric properties [16]. Xiaowei Yin et al. fabricated graphene/ZnO composites [17], and Man He et al. designed TiO_2_/Ti_3_C_2_T_x_/RGO composites [14], achieving good EMW absorption effects. Meanwhile, due to its unique two-dimensional structure, graphene has a high specific surface area, which helps to improve the interface contact between graphene and other components, thus enhancing the interface polarization effect. Jianping He et al. significantly improved the polarization strength and impedance matching performance of Fe_3_O_4_/ graphene composites by introducing the laminated graphene structure between Fe_3_O_4_ nanoparticles. The improved polarization loss strength improves the absorption strength of Fe_3_O_4_/ graphene, and the improved impedance matching performance broadens the effective EMW absorption frequency bandwidth, which made the composites achieve better EMW absorption performance [18]. 

These studies proved that, by adjusting the type and proportion of components, the impedance matching characteristics of composites can be effectively adjusted while maintaining a high level of attenuation loss characteristics, and the effective EMW absorption bandwidth of absorbing material can be significantly widened [19,20,21,22]. In addition, by introducing carbon materials, an efficient conductive network can be formed inside the composites, which also promotes the reduction of the amount of absorbing materials [23,24,25,26]. Therefore, by introducing graphene into OIHP materials, a new type of OIHP/graphene-composite-absorbing material with OIHP as the primary material and graphene as the supplement is expected to reduce the addition amount and realize broadband absorption of EMW.

In this paper, CH_3_NH_3_PbI_3_/graphene composites (MPI/graphene) were prepared using an anti-solvent method. Firstly, the physical phase properties and microstructure of MPI/graphene composites were characterized, and the effect of graphene on the crystallinity and crystalline phase of MPI was investigated. Then, the optimal EMW absorption properties of MPI/graphene composites were determined by adjusting the component ratios of MPI and graphene. Finally, the mechanism of EMW absorption performance of MPI/graphene composites was analyzed to accumulate relevant experience for the composite of MPI and two-dimensional high dielectric materials.

## 2. Materials and Methods

### 2.1. Materials

Graphene was obtained from Nanjing Xianfeng Nano Co., Ltd. (Nanjing, China), while the particle size of graphene was 1–5 μm, the thickness was 1–5 nm, the specific surface area was more significant than 100 m^2^/g, and the electrical conductivity was 1000–1500 S/cm. Lead iodide (PbI_2_, 99%), methylamine iodine (MAI, 99%), Gamma-butyrolactone (γ-GBL, 99%), and anisole (C_7_H_8_O, 99.9%) were supplied by Advanced Election Technology Co., Ltd. (Dalian, China). 

### 2.2. Synthesis of MPI/Graphene Composites

The anti-solvent method prepared MPI/graphene composites (MG) [8]. Firstly, the MAI powder and PbI_2_ powder were dissolved in γ-GBL solution and stirred continuously at 80 °C for 1 h to prepare 0.8 mol/mL of MPI precursor solution. Graphene powder was dispersed in γ-GBL solution at 10 mg/mL concentration for 3 h at 25 °C with continuous stirring. Then, the MPI precursor solution was mixed with the graphene suspension in a specific ratio and stirred continuously at 25 °C for 1 h. The mixing ratio of MPI precursor solution and graphene suspension is shown in Table 1. After the reaction, the mixture of MPI/graphene solution was quickly added to the excessive anisole solution dropwise, and a large number of black suspensions could be observed during the reaction process. After the drip process, the anisole solution was placed in a nitrogen atmosphere for 12 h. Finally, the underlying precipitate in the anisole solution was washed several times by centrifugation with isopropanol and hexane, and the product was dried in a vacuum at 80 °C for 24 h. The resulting black sample is the MPI/graphene composite.

### 2.3. Characterization

The X-ray diffraction (XRD) was carried out with Cu Kα radiation (λ = 1.5406 Å) at 40 kV, and SEM images were obtained in an S-4800 (Hitachi, Tokyo, Japan) machine. Raman spectroscopy (Raman) and steady-state photoluminescence (PL) spectrums were recorded with a Renishaw InVia Basis Raman Spectrometer (Renishaw, London, UK) and an FLS980 Series of Fluorescence Spectrometers, respectively. A precision LCR meter (TH2829C) and current density tester (SDM-200) were used to research the electrical conductivity (EC) of samples. The crystal features were tested by X-ray photoelectron spectroscopy (250XI, Thermo Scientific, Berlin, German). The electromagnetic properties of the pieces were measured by the Agilent PNA N5244A vector network analyzer with the coaxial probe method [27,28], and the electromagnetic parameters of the sample in the range of 2–18 GHz were obtained, including the real (ε′ and μ′) and imaginary (ε″ and μ″) parts of permittivity and permeability. To measure the electromagnetic properties, the samples were uniformly mixed with paraffin in a mass ratio of 2:3 and pressed into coaxial rings, which were toroidal shapes with an outer diameter of 7 mm and an inner diameter of 3.04 mm.

## 3. Results and Discussion

**Figure 1:**
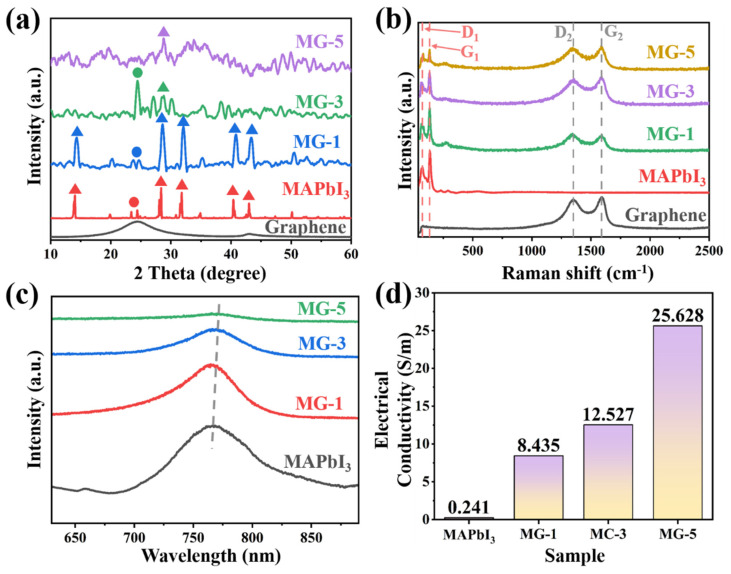
The XRD patterns (**a**), Raman spectrum (**b**), photoluminescence (PL) spectrum (**c**), and electrical conductivity diagram (**d**) of MG composites, MPI crystals, and graphene.

X-ray diffraction analysis technology analyzed MG composites’ phase and crystallinity. For comparison purposes, the XRD patterns of MG composites, MPI crystals, and graphene are all given in Figure 1a. The XRD pattern of MPI crystals shows that the characteristic diffraction peaks of MPI appeared at 2θ = 14.1°, 23.4°, 24.4°, 28.4°, and 31.9°, which corresponds to the lattice plane of (002), (211), (202), (220), and (222), respectively. These diffraction peaks mean that MPI crystals exist in a tetragonal structure [29,30]. For the XRD pattern of the MG-1 sample, five typical characteristic peaks corresponding to the tetragonal phase structure of MPI crystals can be clearly seen, which indicates that MPI in the MG-1 sample still exists in a specific tetragonal phase structure. At this ratio, the addition of graphene did not have a significant impact on the growth crystallization process of MPI. For the XRD pattern of MG-3 samples, with the increase in the proportion of graphene material in MG composites, the characteristic diffraction peaks of corresponding tetragonal-phase MPI crystals disappeared, and the intensity of diffraction peaks at 2θ = 24.4° significantly increased, which indicates that the grain integrity of MPI crystals in MG-3 samples was seriously damaged. There may be many defects and vacancies on the crystal surface. It also can be seen that the characteristic diffraction peaks of MPI crystals cannot be found at the ratio of MG-5 samples. According to the above results, it can be shown that the addition of graphene has an inhibitory effect on the growth and crystallization process of MPI. With the increase in graphene content, the crystallinity of MPI crystals decreased, and the crystals began to appear with vacancies and defects. When graphene was excessive, the crystallization process of MPI was utterly destroyed, and MG composites could not be synthesized. In the XRD patterns of MG samples, the characteristic diffraction peak corresponding to the graphene (2θ = 24.1°) could not be seen. This is because the crystallinity of the graphene material is much lower than that of MPI crystals. Therefore, to analyze the existence form and state of the graphene samples in MG materials, Raman spectra were introduced to further study MG composites.

Figure 1b shows the Raman spectra of MG composites, MPI crystals, and graphene. Two distinct sets of absorption peaks can be seen in the Raman spectrum of the MG composites. The D_1_ and G_1_ peaks at 73.2 cm^−1^ and 135.5 cm^−1^ correspond to the characteristic absorption peaks of MPI crystals, while the D_2_ and G_2_ peaks at 1350.3 cm^−1^ and 1589.4 cm^−1^ correspond to the characteristic absorption peaks of graphene [31,32,33]. The presence of two sets of characteristic peaks demonstrates the existence of MPI and graphene in the MG composites. Meanwhile, the intensity ratio between the D and G peaks also reflects the lattice integrity of the test samples, with an increase in the I_D_/I_G_ ratio, indicating more vacancies and defects in the lattice of that group of test samples. With the increase in graphene content in MG composites, it can be seen that the ratio of I_D1_/I_G1_ increased from 0.61 of MG-1 to 0.85 of MG-3 and then to 0.95 of MG-5. This indicates that with the increase in graphene content, the lattice integrity of MPI in MG composites significantly decreased, which is consistent with the XRD analysis. Compared with the obvious change of I_D1_/I_G1_ values, I_D2_/I_G2_ values remained stable in all proportions (MG-1 = 0.96, MG-3 = 0.94, MG-5 = 0.96). Therefore, it can be inferred that graphene not only existed statically in MG composites but also did not react with MPI crystals without any change in lattice state. 

The steady-state photoluminescence spectra of MG composites are shown in Figure 1c. As a kind of fluorescent material, hybrid perovskite has a very typical characteristic fluorescence peak. As an essential method to analyze the crystallinity of perovskite materials, the PL spectrum was also used to analyze the lattice structure of MPI crystals in MG composites. According to Figure 1c, the intensity of the characteristic fluorescence peak of the MG composites decreased, and the half-peak width narrowed as the graphene content in the MG increased, indicating an increase in the number of vacancies and defects within the MPI crystals. At the same time, a slight rightward shift of the characteristic fluorescence peak can also be observed, suggesting that the disruption of the MPI crystal structure leads to an increase in its band gap, which may result in a shift in the electromagnetic absorption band [34]. Good electrical conductivity is an essential condition for dielectric loss of EMW. From Figure 1d, the electrical conductivity of MPI crystals was low, while introducing graphene material will significantly improve the electrical conductivity of MG composites. This is due to the excellent electrical conductivity of the graphene and the fact that the two-dimensional layer structure of the graphene allows for a larger surface area of contact between the graphene and the MPI, which facilitates the rapid movement of free electrons between the MPI crystals and the MG composites.

**Figure 2:**
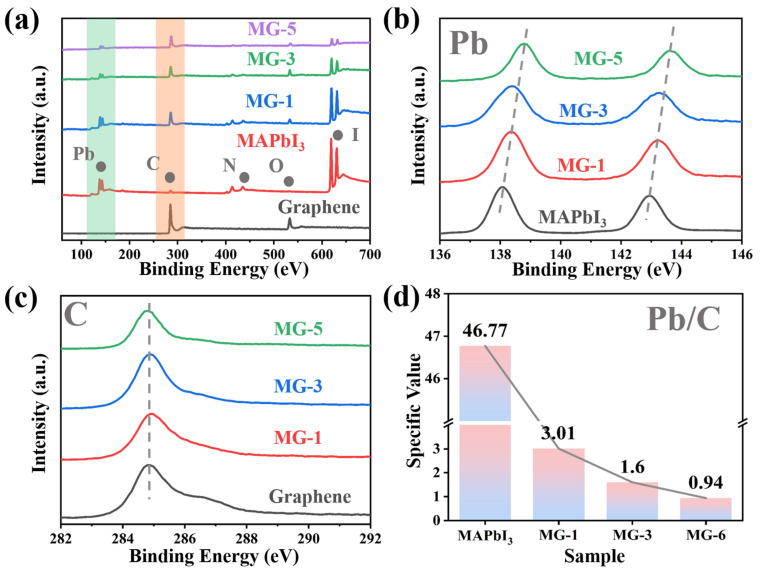
The XPS spectra of MG composites, MPI crystals, and graphene (**a**); the XPS spectra of Pb (**b**) and C (**c**); the Pb/C content ratio of MG composites (**d**).

The XPS survey spectra of MG composites, MPI crystals, and graphene are shown in Figure 2a. Graphene material is mainly composed of C elements. The C peak at 284.8 eV corresponds to the C–C bond in the aromatic ring structure of graphene. In comparison, the O peak at 533.0 eV indicates a small number of oxygen-containing functional groups on the surface or at the edges of the graphene [35]. As for the MPI crystals, two Pb 4f7 and Pb 4f5 peaks were located at 138.0 eV and 143.0 eV, respectively. The peak at 285.0 eV corresponded to C 1s, and the height at 402.0 eV corresponded to N 1s. The 533.0 eV and 619.0 eV peaks corresponded to O 1s and I 3d, respectively [36,37]. At the same time, the characteristic peaks of the above elements can also be observed in the XPS spectra of MG composites, indicating the stable existence of MPI crystals and graphene in MG composites. However, it should be noted that with the increase in graphene content, except for the characteristic peaks of C and O elements, the intensity of other elemental peaks decreased significantly. On the one hand, due to the influence of graphene, the crystal structure of MPI crystals was destroyed and decomposed. On the other hand, XPS analysis had limited sample penetration, typically to a depth of 5–10 nm. The reduced intensity of the characteristic elemental peaks for Pb and I reflected the increased graphene content wrapped around the outer layers of the MPI crystals, which the electron beam from the XPS analyzer could not accurately detect.

Figure 2b shows the XPS spectra of the Pb element. It can be seen that with the increase in graphene content, compared with MPI crystals, the characteristic peaks of Pb 4f_7_ and Pb 4f_5_ in MG composites shifted significantly to the right, and the degree of rightward shift gradually increased. According to Figure 2c, the characteristic peak of element C was more consistently located near 285.0 eV, which is further evidence of the high stability of graphene in MG composites, where the lattice morphology and surface structure were not significantly affected by the MPI crystals. The XPS energy spectrum and date of C1s sub-peak of MG-3 have are provided in Figure S1 and Table S1. As shown in Figure 2d, the Pb/C content ratio of MPI crystal was 46.771, and that of MG composite decreased from 3.012 (MG-1) to 0.936 (MG-5). This proved that the content of graphene in MG composites was steadily increasing. The MG composites with different components were successfully prepared by the anti-solvent method.

**Figure 3:**
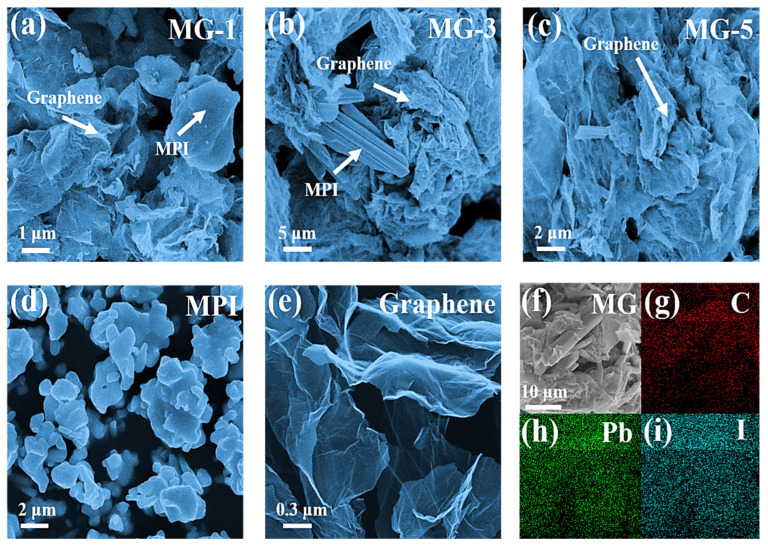
The SEM images of MG-1 (**a**), MG-3 (**b**), MG-5 (**c**), the MPI crystals (**d**), and graphene (**e**); the elemental mapping images of MG-3 (**f**), C (**g**), Pb (**h**), and I (**i**).

Figure 3 shows the SEM images of the MPI crystals, graphene, MG-1, MG-3, and MG-5 composites. In Figure 3d, the individual MPI crystals can be seen as three-dimensional blocks with a size of about 2–3 μm. As shown in Figure 3e, the graphene exhibited a distinctive two-dimensional layered structure with a large specific surface area, a monolayer thickness of less than 5 nm, and layer-to-layer folds [38]. According to Figure 3a–c, in MG-1, it can be seen that the MPI crystals still maintained a three-dimensional massive structure and had an increased grain size compared to the MPI crystals. The graphene material was distributed around the MPI crystals and had less contact area with the MPI crystals. A change in the morphology of the MPI crystals can be seen in MG-2, with a shift from a three-dimensional bulk to a one-dimensional rod structure. At the same time, the MPI micron rod was wrapped by the graphene, which indicates that the contact area of the graphene and MPI crystals significantly increased at this component ratio. No noticeable MPI crystals were observed in MG-3, while the graphene material was abundantly distributed. This confirmed the XPS analysis: the MPI crystals in MG-5 were partially destroyed, and graphene was wholly wrapped around some of the defective MPI crystals. The elemental mapping images of MG-3 are shown in Figure 3f–i. The C, Pb, and I elements were uniformly distributed in MG-3, which means that the MPI crystals and graphene were successfully combined to form a stable composite material. This is consistent with the XRD, Raman, XPS analysis results, and verifies the feasibility of the anti-solvent method for the preparation of MG composites.

**Figure 4:**
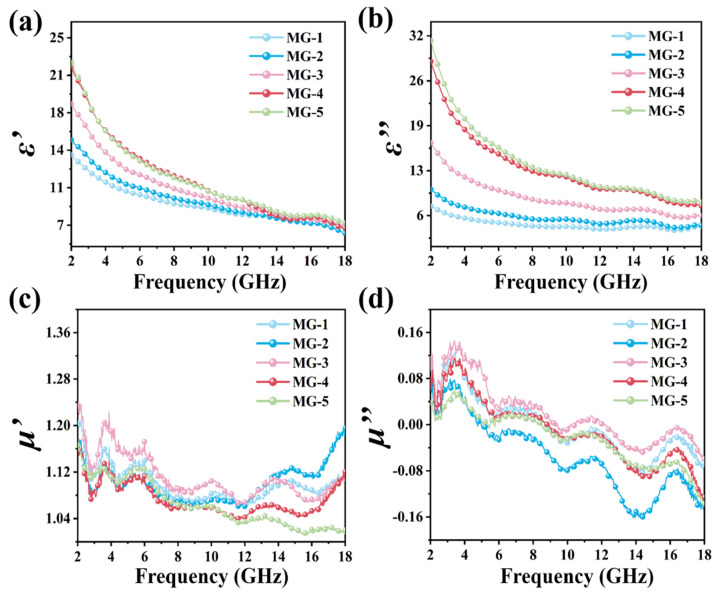
Complex permittivity and permeability of the MG composites: $\varepsilon^{\prime }\;$(**a**), $\varepsilon^{\prime \prime }\;$(**b**), $\mu^{\prime }\;$(**c**), and $\mu^{\prime \prime }\;$(**d**).

To further explore the dissipative ability of composite materials on EMW, the electromagnetic parameters of MPI crystals, graphene, and MG composites within 2–18 GHz were studied under the coaxial-ring doping amount of 40 wt%. The relevant test results are shown in Figure 4 and Figure S2, respectively. The electromagnetic parameters of MG composites with different component proportions are given in Figure 4. On the one hand, with the increase in graphene content in MG composites, the $\varepsilon^{\prime }$ value and $\varepsilon^{\prime \prime }$ The value of MG composites increased regularly. According to the equation ($\varepsilon^{\prime \prime }\approx 1/\pi \varepsilon_{0}\rho f$) [39], there was a negative correlation between the resistivity (*ρ*) and $\varepsilon^{\prime \prime }$, the increase in the $\varepsilon^{\prime \prime }$ value indicates a decrease in resistivity and an increase in electrical conductivity of the MG samples, suggesting that the addition of graphene enhanced the electrical conductivity loss of the MG composites. On the other hand, the $\varepsilon^{\prime }$ value and $\varepsilon^{\prime \prime }$ value of MG composites affected by the graphene also showed a trend of decreasing values with the increase in EMW frequency in the range of 2–18 GHz. In comparison, the $\mu^{\prime }$ value of MG composites at each component ratio was approximately 1.08 and the $\mu^{\prime \prime }$ value was approximately 0, suggesting that MG composites have strong dissipation ability to the electric energy carried by the incident EMW but weak dissipation to the magnetic energy. 

**Figure 5:**
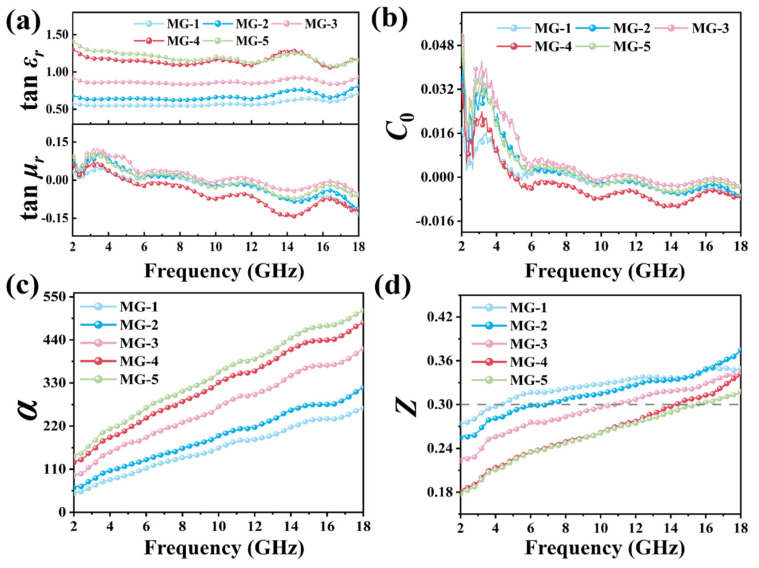
Dielectric loss tangent and magnetic loss tangent (**a**), C_0_ (**b**), the attenuation constant (**c**), and intrinsic impedance ratio (**d**) of the MG composites.

The dielectric loss tangent ($tan\varepsilon_{r}$) and magnetic loss tangent ($tan\mu_{r}$) are shown in Figure 5, which can be calculated as follows [40]:

(1) $$tan\varepsilon_{r}=\frac{\varepsilon^{\prime \prime }}{\varepsilon^{\prime }}$$

(2) $$tan\mu_{r}=\frac{\mu^{\prime \prime }}{\mu^{\prime }}$$

The $tan\varepsilon_{r}$ and $tan\mu_{r}$ represent the dissipation capacity of a material to electric energy and magnetic energy carried by incident EMW, respectively. In Figure 5a, the addition of graphene improved the dissipation ability of MG composites to electric energy while the dissipation ability of magnetic energy was not significantly affected. An analysis of the magnetic loss properties of MG can be obtained in supporting information. Figure 5b is the eddy current loss coefficient of MG composites. The study of magnetic loss characteristics of MG composites can be obtained from Supporting Information.

The attenuation loss constant (α) reflects the ability of a material to attenuate the EMW entering its interior, and previous studies demonstrated that when the attenuation loss constant is higher than 100, the material can achieve effective attenuation of the incident EMW. The attenuation loss constant can be calculated as follows [41]:

(3) $$\alpha =\frac{\sqrt{2}\pi f}{c}\times \sqrt{\left(\mu^{\prime \prime }\varepsilon^{\prime \prime }-\mu^{\prime }\varepsilon^{\prime }\right)+\sqrt{{\left(\mu^{\prime \prime }\varepsilon^{\prime \prime }-\mu^{\prime }\varepsilon^{\prime }\right)}^{2}+{\left(\mu^{\prime }\varepsilon^{\prime \prime }+\mu^{\prime \prime }\varepsilon^{\prime }\right)}^{2}}}$$

The intrinsic impedance ratio *Z* reflects the difficulty of EMW entering the material’s interior. It was found that when the intrinsic impedance ratio of the material is more significant than 0.3, EMW can enter the material’s interior in large quantities. The inherent impedance ratio *Z* can be calculated as follows [42]:

(4) $$Z=Z_{r}/Z_{0}$$

(5) $$Z_{r}=Z_{0}\sqrt{\left|\mu_{r}\right|/\left|\varepsilon_{r}\right|}$$

As shown in Figure 5c, the attenuation loss coefficient of MG composites at each component ratio was relatively high. While the attenuation loss coefficient of some composites at the low-frequency band (2–5 GHz) was lower than 100, the remaining attenuation loss coefficients were all over 100, which indicates that MG composites at each component ratio can achieve good attenuation loss characteristics for incident EMW. In Figure 5d, the impedance matching ratio of MG-1 and MG-2 are relatively high and higher than 0.3 in the 6–18 GHz range. This indicates that only MG-1 and MG-2 composites allow many EMW to enter the interior of the absorbing materials. Graphene content (MG-3, MG-4, MG-5) that is too high will lead to many EMW reflected or refracted on the surface of the absorbing materials, which can not be effectively attenuated. It can be inferred that the absorption performance of MG materials is regulated and affected by the intrinsic impedance ratio, and it is expected that MG-1 and MG-2 composites will show better EMW absorption performance. Combined with the analysis of the attenuation loss coefficient and intrinsic impedance ratio of MG samples, the ability of MG materials to attenuate and lose EMW gradually increased as the content of graphene increased, and the difficulty of EMW being able to enter inside the absorbing material gradually increased. This shows that adding graphene improved the polarization loss strength and conductance loss strength inside MG materials. And the MPI crystal, as the center of electric polarization composite with graphene, can also effectively adjust the impedance matching of the composite material, which is conducive to improving the absorption performance.

**Figure 6:**
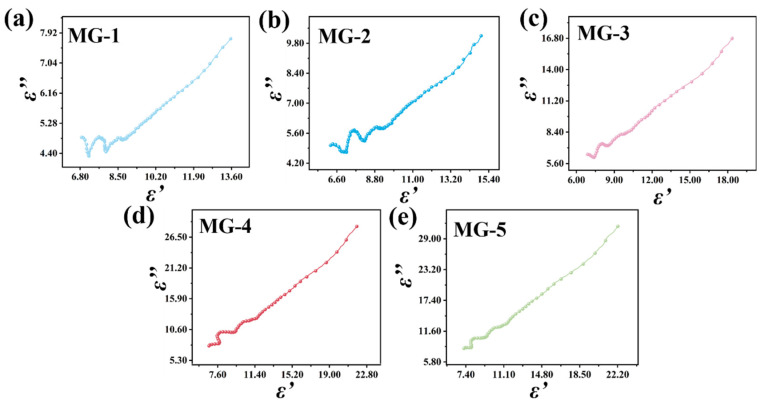
Cole–Cole plots of MG composites: MG-1 (**a**), MG-2 (**b**), MG-3 (**c**), MG-4 (**d**), MG-5 (**e**).

In order to analyze the polarization relaxation phenomenon of the dielectric loss-absorbing materials, the Debye relaxation process of MG composites with different component ratios is described by the Cole–Cole diagram. Cole–Cole diagrams are calculated and plotted according to the following Debye relaxation formula [41]:

(6) $${\left(\varepsilon^{\prime }-\frac{\varepsilon_{s}+\varepsilon_{\infty }}{2}\right)}^{2}+{\left(\varepsilon^{\prime \prime }\right)}^{2}={\left(\frac{\varepsilon_{s}-\varepsilon_{\infty }}{2}\right)}^{2}$$

where $\varepsilon_{s}$ is the static dielectric constant and $\varepsilon_{\infty }$ is the relative dielectric constant of the high-frequency limit. A semicircle equation with $(\varepsilon_{s}-\varepsilon_{\infty })/2$ as the radius and ($0,(\varepsilon_{s}+\varepsilon_{\infty })/2$) as the circle’s center can be established from the Debye relaxation equation, and the resulting circle is known as a Cole–Cole semicircle. Usually, each semicircle represents a relaxation process within the material, and the number of semicircles represents the intensity of the relaxation polarization. Figure 6 shows Cole–Cole semicircular graphs of MG composites with different component ratios, and these Cole–Cole graphs show similar trends. In the high-frequency band, the five samples all have two semi-circles, but with the increase in graphene content, the circle’s radius gradually decreases. On the one hand, this phenomenon shows that MG samples in the high-frequency band will produce pronounced relaxation polarization under the electromagnetic field, which is conducive to improving the dielectric loss strength of materials. On the other hand, it also shows that excessive graphene will cause the relaxation polarization intensity to decrease. When the radius of the Cole–Cole circle decreases and is close to a straight line, the electrical conductivity loss dominates. This conclusion is also applicable to the positive correlation between the real and imaginary parts of the dielectric constant of MG samples in the low-frequency band, indicating that the electrical conductivity loss of MG samples is the dominant form of low-frequency EMW loss, with the Debye relaxation polarization making a more minor contribution to the dielectric loss intensity. The irregularity of the Cole–Cole semicircle suggests that high-frequency EMW will lead to Debye relaxation polarization and multiple polarization loss processes, including interface polarization, electron polarization, and dipole polarization.

**Figure 7:**
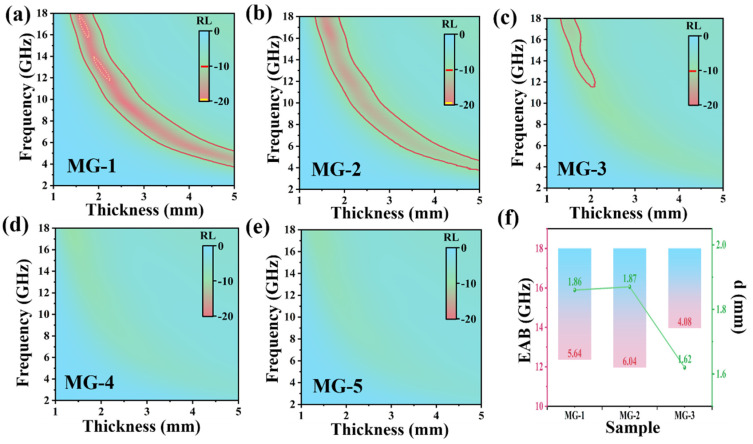
Reflection loss curves of MG-1 (**a**), MG-2 (**b**), MG-3 (**c**), MG-4 (**d**), and MG-5 (**e**); the EAB of MG composites with corresponding absorption thicknesses (**f**).

Based on the transmission line theory, the value of reflection loss (RL) can be calculated by the following equations [43,44]:

(7) $$Z_{in}=Z_{0}\sqrt{\frac{\mu_{r}}{\varepsilon_{r}}}tanh\left(j\frac{2\pi fd}{c}\sqrt{\mu_{r}\varepsilon_{r}}\right)$$

(8) $$RL\left(dB\right)=20lg\left|\frac{Z_{in}-Z_{0}}{Z_{in}+Z_{0}}\right|$$

where $Z_{in}$ is the normalized input impedance corresponding to the thickness of the absorber, d is the thickness of the absorber, and h is the Planck constant. Figure 7 shows the RL of MG samples in the 2–18 GHz range when the doping amount is 40 wt%, and the thickness is 1 mm to 5 mm. In the figure, the area in the red line indicates that the RL is less than −10 dB, and the yellow dotted line indicates that the RL is less than −20 dB. The area where the RL is less than −10 dB is usually called the effective electromagnetic absorption band (EAB) of the absorbing material. According to Figure 7, the overall EMW absorption performance of MG materials shows a decreasing trend as the graphene content increases. Figure 7f shows the EAB of MG-1, MG-2, and MG-3 composites with corresponding absorption thicknesses. The EAB of MG-2 sample reaches 6.04 GHz (11.96–18.00 GHz) at a 1.87 mm absorber thickness, which is wider than MG-1 and MG-3. This is consistent with the conclusion obtained from the analysis of the attenuation loss coefficient and intrinsic impedance ratio. MG-3 sample shows better absorbing performance in the high-frequency range with the EAB of 4.08 GHz at 1.62 mm thickness. For MG-4 and MG-5, there is no significant EAB in the 2–18 GHz range due to the large amount of graphene resulting in samples with intrinsic impedance ratios well below 0.3 and a large amount of EM waves being reflected at the absorber surface. Combined with the characterization results of MG materials, it can be seen that when the crystallinity of MPI crystals in the material is very low, the incident EMW cannot be effectively absorbed by polarization. MG’s impedance matching performance (IMP) can be obtained in supporting information.

**Figure 8:**
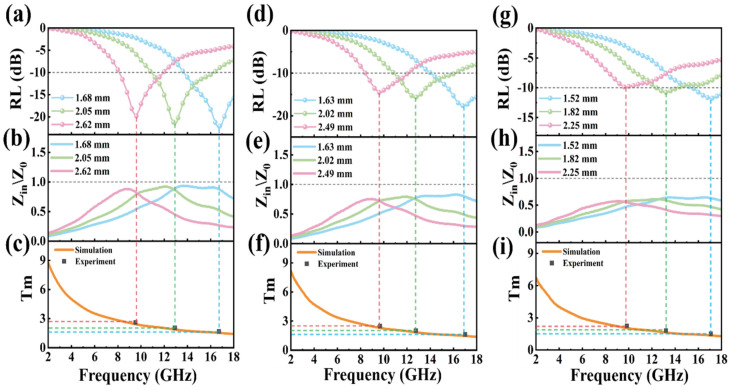
MG-1: the correlation between the maximum RL (**a**), normalized input impedance (**b**), and the absorption thickness (**c**); MG-2: the correlation between the maximum RL (**d**), normalized input impedance (**e**), and the absorption thickness (**f**); MG-3: the correlation between the maximum RL (**g**), normalized input impedance (**h**), and the absorption thickness (**i**).

EAB and EMW absorption intensity are two key indexes to evaluate the properties of absorbing materials. Based on the outstanding EAB properties of MG-1, MG-2, and MG-3 samples, the absorption intensity of the three samples is further studied in Figure 8. The correlation between maximum reflection loss (RL), normalized input impedance ($Z_{d}$), and the absorption thickness ($T_{m}$) is shown in Figure 8. The absorption thickness ($T_{m}$) can be calculated by [39]:

(9) $$T_{m}=\frac{n\lambda }{4}=\frac{nc}{\left(4f_{m}\sqrt{\left|\mu_{r}\right|\left|\varepsilon_{r}\right|}\right)},\left(n=1,3,5,\ldots \right)$$

MG-1’s maximum RL value of −22.689 dB at 16.72 GHz is found at a thickness of 1.68 mm. The second largest RL value of −21.878 dB appears at 12.92 GHz, corresponding to a thickness of 2.05 mm. When the absorption thickness is 2.62 mm, the RL value of MG-1 at 19.52 GHz is −19.929 dB. Combined with Figure 8b, it can be found that when the normalized input impedance value at the peak reflection loss frequency is closer to 1.0, the RL intensity of the absorbing material is higher. This shows that good impedance matching is beneficial to enhance the EMW absorption performance of the material. In addition, as the thickness of the material increases, the maximum RL peak of the MG-1 material moves towards lower frequencies, which the quarter-wavelength theory can explain. The orange line in Figure 8c represents the t_m_ simulation curve of MG-1 material, and the gray box represents the thickness of the absorber corresponding to the maximum reflection loss. The grey squares are evenly distributed around the simulation curve, indicating that the absorption mechanism of MG-1 material conforms to the quarter-wavelength theory. The conclusions in the MG-1 material can also explain the relevant phenomena in the MG-2 and MG-3 materials. The EMW absorption properties of some MG samples are given in Table 2. Collectively, the MG-1 and MG-2 samples exhibited superior EMW absorption properties.

The EMW absorption properties of MG materials result from the combined effect of MPI crystals and graphene, which improves the impedance matching of MG samples by adjusting the ratio of the two components. As a typical dielectric loss absorbing material, graphene wraps and connects several MPI crystals in series, which creates conditions for free electrons to move freely between the MPI crystals, forming a large number of field-induced microcurrents inside the MG materials under an alternating electric field, which effectively converts electromagnetic energy into heat by electrical conductivity losses. In addition, the polarization loss effect is sufficiently enhanced to improve the EMW absorption performance of MG composites substantially: Firstly, the addition of graphene disrupts the crystal structural integrity of the MPI, generating a large number of surface defects and vacancies and enhancing the space charge polarization effect of the MG materials significantly under the action of external electric field. Secondly, because the MPI crystals and graphene are different in dielectric constant and electrical conductivity, this will lead to charge accumulation at the interface of the two dielectric materials, resulting in an interface polarization effect.

## 4. Conclusions

In this paper, MPI crystals and graphene were successfully compounded by an anti-solvent method to fabricate MG composites with different component proportions. The phase composition, lattice morphology, and micro-morphology of MG materials with different proportions were analyzed by XRD, Raman, PL, and SEM. The EMW absorption performance of MG materials was fully investigated in terms of the type of EMW-absorbing materials, absorption mechanism, effective EMW absorption bandwidth, and reflection loss strength. It was found that for MG composites, MG materials at MG-1 and MG-2 ratios exhibited better EMW absorption performance than other ratios. This work demonstrates that introducing two-dimensional carbon materials plays an important role in broadening the effective EMW absorption band of MPI crystals and reducing the amount of EMW-absorbing materials added within the absorber.

## Figures and Tables

**Table 1:** The mixing ratio of MPI precursor solution and graphene suspension.

MPI Precursor Solution (mL)	Graphene Suspension (mL)	MPI/Graphene	Sample Number
0.806	2.4	24:1	MG-1
0.806	3.75	16:1	MG-2
0.806	4.95	12:1	MG-3
0.806	7.5	8:1	MG-4
0.806	9.9	6:1	MG-5

**Table 2:** EMW absorption properties of MG samples.

Sample	Point	|RL| (dB)	d (mm)	f (GHz)
MG-1	$m_{1}$	22.69	1.68	16.72
	$m_{2}$	21.88	2.05	12.92
	$m_{3}$	19.93	2.62	9.52
MG-2	$m_{1}$	17.68	1.63	17.00
	$m_{2}$	15.74	2.02	12.76
	$m_{3}$	14.53	2.49	9.68
MG-3	$m_{1}$	11.75	1.52	17.08
	$m_{2}$	10.78	1.82	13.20
	$m_{3}$	9.97	2.25	9.84

## Data Availability

Data are contained within the article or supplementary material. The data presented in this study are available in article and supplementary material.

## Supplementary Materials

The following supporting information can be downloaded at: https://www.mdpi.com/article/10.3390/mi14081611/s1, Figure S1. XPS energy spectrum of C1s sub peak of MG-3; Figure S2. Complex permittivity and permeability of the MPI crystals and graphene: $\varepsilon^{\prime }$ (a), $\varepsilon^{\prime \prime }$ (b), $\mu^{\prime }$ (c), and $\mu^{\prime \prime }$ (d); Figure S3. The impedance matching performance of the MG samples: MG-1 (a), MG-2 (b), MG-3 (c), MG-4 (d), and MG-5 (e). Table S1. The date of C1s sub peak of MG-3. References [4,45] are cited in the supplementary materials.
