# Supplementary material for: A Highly Efficient Electromagnetic Wave Absorption System with Graphene Embedded in Hybrid Perovskite
Source: Micromachines (Basel). 2023 Aug 16;14(8):1611. doi: 10.3390/mi14081611 (PMC10456661; doi:10.3390/mi14081611)
Supplement: Supplementary file 1 [file micromachines-14-01611-s001.zip › micromachines-2558083-SI.pdf]

A Highly Efficient Electromagnetic Wave Absorption System with Graphene Embedded in Hybrid Perovskite

Haitao Yu [1], Hui Liu [2], Yao Yao [3], Ziming Xiong [3], Lei Gao [4], Zhiqian Yang [3], Wenke Zhou [3,5,*] and Zhi Zhang [4,*]

1 Field Engineering College, Army Engineering University of PLA, Nanjing 210007, China
2 Unit of 32399 of PLA, Nanjing 211131, China
3 State Key Laboratory for Disaster Prevention & Mitigation of Explosion & Impact, Army Engineering University of PLA, Nanjing 210007, China
4 Position Engineering Research Office, Army Engineering University of PLA, Nanjing 210007, China
5 Electromagnetic Environmental Effects Laboratory, Army Engineering University of PLA, Nanjing 210007, China
* Correspondence: zhou.w.k@163.com (W.Z.); zhangnjjn@163.com (Z.Z.)

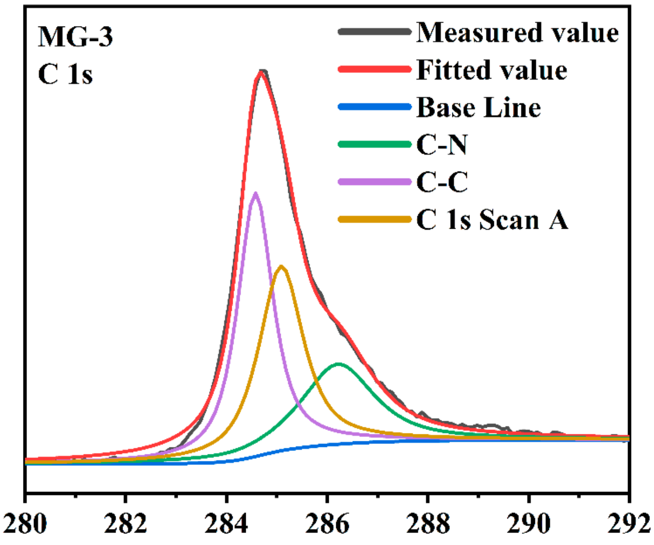

Figure S1. XPS energy spectrum of C1s sub peak of MG-3

Table S1. The date of C1s sub peak of MG-3

Type	Covalent bond	Area	Ratio
MPI	C-N	16606.35	0.61
	C 1s Scan A	23586.62	
Graphene	C-C	26765.94	0.39

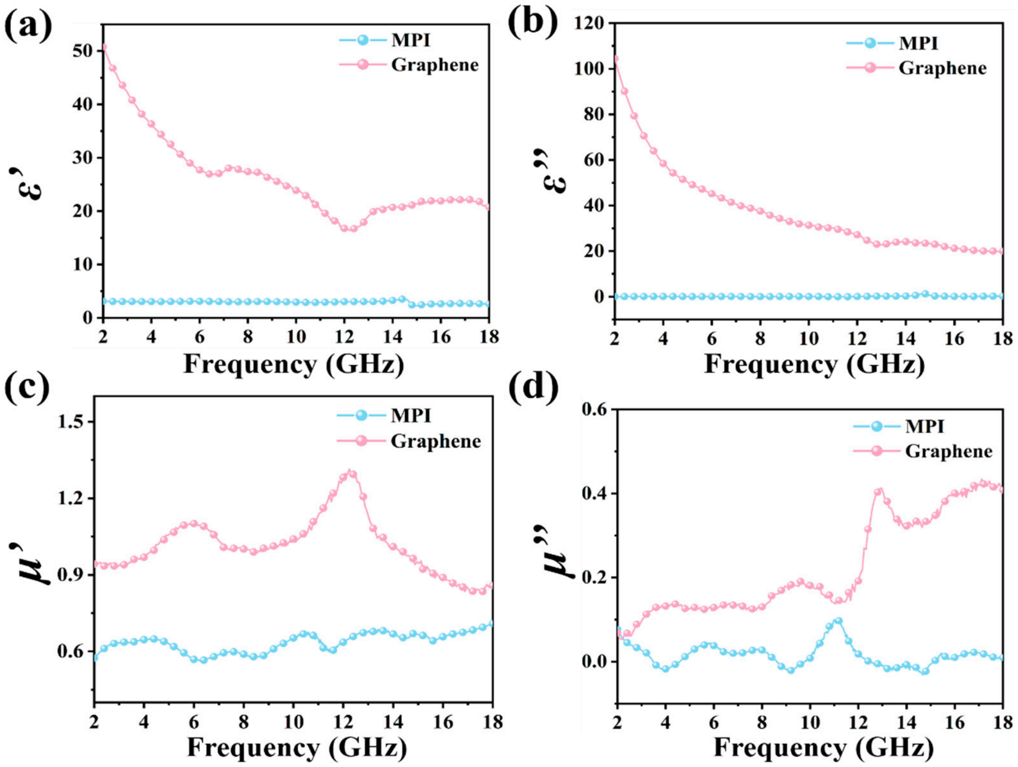

Figure S2. Complex permittivity and permeability of the MPI crystals and graphene: ε'(a), ε''(b), μ'(c), and μ''(d)

The XPS energy spectrum and date of C1s sub peak of MG-3 have been provided in Figure S1 and Table S1. It can be seen that the proportion of C-N bond and satellite peak representing MPI was about 0.6, and the proportion of C-C bond representing graphene was 0.4. This is different from the mass ratio of MPI: graphene=12:1, but because XPS mainly tests the elemental composition at 5-10 nm of the sample surface, this result shows that graphene successfully wrapped MPI crystals, which not only increases the interface polarization performance of MG, but also facilitates the play of conductance loss.

In Figure S2, for the graphene, the real part of the complex permittivity (ε') and the imaginary part of the complex permittivity (ε'') decreased with the increase of the incidence frequency of EMW. However, the ε' value and ε'' value of MPI crystals are less affected by the frequency variation of incident EMW, and only exhibit partial oscillations in the range of 14-16 GHz. This shows that graphene have different dissipation capacity for different frequency EMW, while MPI crystals only have attenuation loss for specific frequency EMW. And the ε' value and ε'' value of graphene are much higher than that of the MPI crystal, which indicates that graphene have a much higher capacity to store and dissipate the electrical energy carried by incident EMW than MPI crystals. As for MPI crystals, the real part of the permeability (μ') and the imaginary part of permeability (μ'') maintain around 0.6 and 0 in the range of 2-18 GHz, which indicates that MPI crystals do not have magnetic loss characteristics. And the slight resonance peaks in the 10-12 GHz

range of the μ'' value curve of MPI crystals may be caused by the natural resonance of atoms in the MPI crystals, which will not cause significant magnetic loss to incident EMW. As a two-dimensional material composed of carbon elements, the graphene do not have magnetic loss characteristics and the fluctuations in the magnetic permeability curves are due to the residual Fe element during the physical preparation of graphene.

Figure 5b is the eddy current loss coefficient of MG composites, which is used to analyze the influence of slight magnetic loss brought by graphene on the wave-absorbing performance. the eddy current loss coefficient can be calculated as follows[1]:

$$C_0 = \frac{\mu''}{\sqrt{\mu'}f} \tag{S1}$$

In general, the magnetic loss of materials mainly comes from hysteresis loss, domain wall resonance, eddy current loss, natural resonance and exchange resonance. The magnetic loss of materials mainly comes from hysteresis loss, domain wall resonance, eddy current loss, natural resonance and exchange resonance. Domain wall resonances usually occur in the MHz frequency range. The hysteresis loss is proportional to the hysteresis loop and can be ignored under weak magnetic field. Natural resonance is the energy loss caused by natural oscillation of a crystal in a magnetic field. Exchange resonance refers to the energy loss caused by vibration caused by the interaction of electric and magnetic fields. The eddy current loss can be judged according to the eddy current loss coefficient of the material. According to the skin effect criterion, if the magnetic loss is due only to eddy current loss, C_0 should be a constant. From Figure 5b, the C_0 curves show a significant change trend with the increase of frequency, so there is no eddy current loss in MG composites, which means that the slight magnetic loss characteristics of MG composites are mainly from natural resonance and exchange resonance.

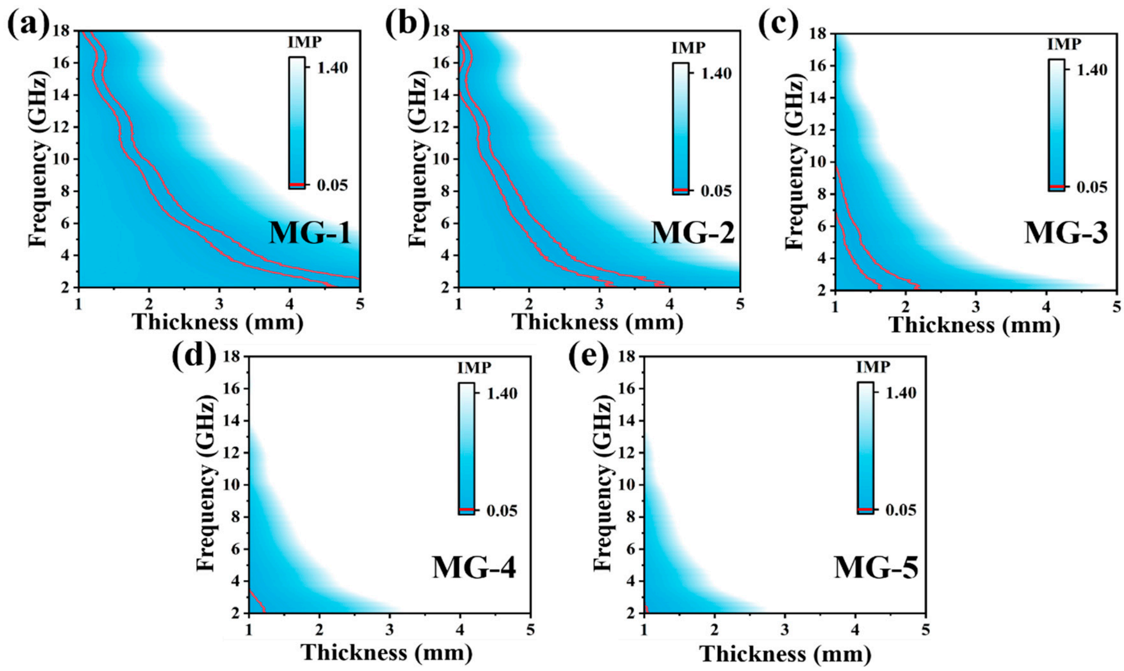

Figure S3. The impedance matching performance of the MG samples: MG-1(a), MG-2(b), MG-3(c), MG-4(d), and MG-5(e)

Impedance matching performance (IMP) of absorbing material is an important index to evaluate its reflection loss performance, which can be calculated as follows[2]:

$$\begin{cases} IMP = \left|\sinh^2(Kfd) - M\right| \\ K = \dfrac{4\pi\sqrt{\mu'\varepsilon'}}{c}\dfrac{\sin\left[(\delta_\varepsilon+\delta_\mu)/2\right]}{\cos\delta_\varepsilon\cos\delta_\mu} \\ M = \left(4\mu'\varepsilon'\cos\delta_\varepsilon\cos\delta_\mu\right)\left[\left(\mu'\cos\delta_\varepsilon-\varepsilon'\cos\delta_\mu\right)^2+\left(\tan\dfrac{\delta_\mu-\delta_\varepsilon}{2}\right)^2\left(\mu'\cos\delta_\varepsilon+\varepsilon'\cos\delta_\mu\right)^2\right]^{-1} \\ \delta_\varepsilon = \arctan\left(\dfrac{\varepsilon''}{\varepsilon'}\right) \\ \delta_\mu = \arctan\left(\dfrac{\mu''}{\mu'}\right) \end{cases}$$

（S2）

Figure S3 shows the impedance matching performance of the MG samples, where the values within the red line are less than 0.05 and the values within the white area are more than 1.5. Usually, the smaller the value of IMP, the more outstanding the EMW absorption properties exhibited by the material. From Figure S3, it can be seen that as the graphene content increases, the IMP values of the MG samples also tend to increase and the corresponding reflection loss performance decreases. This further verifies the

validity of the above analysis of the reflection loss performance of the MG samples.

References

[1] Zhao B, Shao G, Fan B, et al. Facile preparation and enhanced microwave absorption properties of core-shell composite spheres composited of Ni cores and TiO2 shells [J]. Physical chemistry chemical physics : PCCP, 2015, 17(14): 8802-10.

[2] Wang F, Gu W, Chen J, et al. The point defect and electronic structure of K doped LaCo0.9Fe0.1O3 perovskite with enhanced microwave absorbing ability [J]. Nano Research, 2021.
